# Antimicrobial resistance and machine learning: past, present, and future

**DOI:** 10.3389/fmicb.2023.1179312

**Published:** 2023-05-26

**Authors:** Faiza Farhat, Md Tanwir Athar, Sultan Ahmad, Dag Øivind Madsen, Shahab Saquib Sohail

**Affiliations:** ^1^Department of Zoology, Aligarh Muslim University, Aligarh, India; ^2^Department of Pharmacognosy and Pharmaceutical Chemistry, College of Dentistry and Pharmacy, Buraydah Colleges, Buraydah, Al-Qassim, Saudi Arabia; ^3^Department of Computer Science, College of Computer Engineering and Sciences, Prince Sattam bin Abdulaziz University, Al-Kharj, Saudi Arabia; ^4^Department of Computer Science and Engineering, University Center for Research and Development (UCRD), Chandigarh University, Mohali, Punjab, India; ^5^School of Business, University of South-Eastern Norway, Hønefoss, Norway; ^6^Department of Computer Science and Engineering, Jamia Hamdard University, New Delhi, India

**Keywords:** antimicrobial resistance, antibiotic resistance, machine learning, deep learning, bibliometric analysis, healthcare

## Abstract

Machine learning has become ubiquitous across all industries, including the relatively new application of predicting antimicrobial resistance. As the first bibliometric review in this field, we expect it to inspire further research in this area. The review employs standard bibliometric indicators such as article count, citation count, and the Hirsch index (H-index) to evaluate the relevance and impact of the leading countries, organizations, journals, and authors in this field. VOSviewer and Biblioshiny programs are utilized to analyze citation and co-citation networks, collaboration networks, keyword co-occurrence, and trend analysis. The United States has the highest contribution with 254 articles, accounting for over 37.57% of the total corpus, followed by China (103) and the United Kingdom (78). Among 58 publishers, the top four publishers account for 45% of the publications, with Elsevier leading with 15% of the publications, followed by Springer Nature (12%), MDPI, and Frontiers Media SA with 9% each. Frontiers in Microbiology is the most frequent publication source (33 articles), followed by Scientific Reports (29 articles), PLoS One (17 articles), and Antibiotics (16 articles). The study reveals a substantial increase in research and publications on the use of machine learning to predict antibiotic resistance. Recent research has focused on developing advanced machine learning algorithms that can accurately forecast antibiotic resistance, and a range of algorithms are now being used to address this issue.

## 1. Introduction

Since Fleming’s ground breaking discovery of the first antibiotic, Penicillin, in 1928, a new era of medicine emerged, leading to the development of other antibiotics that are now commonly used to combat bacterial infections. Nevertheless, the recurring use of these antibiotics has resulted in the emergence of drug-resistant bacteria, posing a major global threat. In many cases, clinicians must administer broad-spectrum antibiotics based only on symptoms, or wait for lengthy laboratory test results before providing the appropriate treatment. These antibiotic-resistant bacteria are becoming more prevalent every day, leading to a significant public health issue and placing an immense financial burden on the healthcare system. Additionally, research suggests that the recent COVID-19 pandemic has led to an increase in antimicrobial resistance (AMR) due to the widespread use of disinfectants and antibiotics (Wilson et al., 2020). The World Health Organization (WHO) and the US Centers for Disease Control (CDC) estimate that there are at least 2 million cases of antibiotic-resistant infections globally each year, with 23,000 of them resulting in fatalities, and the global cost of medical care ranging from $20 to $35 billion (Centers for Disease Control and Prevention, 2013). By 2050, the yearly death toll from infections brought on by bacteria resistant to antibiotics would reach 10 million, exceeding the annual death toll from cancer (Murray et al., 2022). A 2014 study found that minor bacterial infections during hip replacement surgery could increase the fatality risk by as much as 30% (Berstock et al., 2014).

To reduce drug abuse, it is essential to quickly and precisely identify strains that are resistant to antibiotics. Detecting antimicrobial resistant (AMR) infections is often a time-consuming and tedious process because some organisms are exact copies of each other or cannot be grown in the lab. Due to advancements in computer-aided drug design and novel *in silico* methods, the creation of antibacterial drugs recently underwent a paradigm shift. Recent studies have demonstrated the efficacy of machine learning methods in predicting AMR in a variety of bacterial strains (Liu et al., 2020). Additionally, it would be extremely time-efficient if machine learning algorithms could be used instead of traditional culturing studies to identify the various mechanisms underlying antimicrobial resistance, such as efflux pumps, target modifications, and enzymatic inactivation, and predict resistance in bacterial strains. After being trained on whole-genome sequencing, a number of machine-learning algorithms, including support vector machines (SVM), logistic regression models (LR), and random forests (RF), have been shown to demonstrate excellent accuracy for predicting AMR (Yang et al., 2018; Liu et al., 2020). Deep learning algorithms’ effectiveness in predicting new antibiotics, AMR genes, and AMR peptides has also recently been proven (Arango-Argoty et al., 2018; Stokes et al., 2020).

In addition to empirical research, review articles, including concise reviews and systematic reviews, have recently been published (Arango-Argoty et al., 2018; Weis et al., 2020; Li et al., 2021; Lv et al., 2021; Kim et al., 2022; Popa et al., 2022), but no bibliometric analysis has been conducted on this specific topic. This study offers a quantitative perspective based on bibliometric data in light of the significance of the research on machine learning uses in AMR. The researcher in this field would be able to choose the research direction for potential future projects with the help of a thorough bibliometric analysis of the machine learning applications used in AMR because the bibliographic study not only highlights the most illustrious researchers, institutions, and countries in a particular field, but also represents the most popular research topics in that field (Yu et al., 2020). Through an analysis of collaborations between authors, institutions, and countries, it is possible to establish a bibliometric and intellectual foundation for a specific area of interest (Farhat et al., 2023). According to our research, this is the first bibliometric analysis on the topic that we are aware of, so we hope that it will encourage researchers to think about fresh directions for their future work in the field. To achieve the aim of the study, research questions were prepared with clear objectives as follows:

**Table:** 

	Research question	Objective
RQ1	Which authors lead the research on machine learning applications in AMR and what are their collaborating network?	To identify the most prolific authors and their collaborating network
RQ2	Which articles are cited the most and which journals are publishing the most on the prediction of AMR using machine learning algorithms?	To identify the most cited publication and the most contributing journals publishing in the field
RQ3	Which organizations and countries contribute most to the scientific production?	To identify the organizations and countries mostly working on the subject
RQ4	What are the trending keywords and which keywords are most cited in the literature on the use of machine learning in AMR identification or prediction?	To identify the trending topics and keywords in the related research filed
RQ5	What is the significant research works going on related to AMR in the view of machine learning applications?	To know the type of research, methods applied, applications and results obtained

## 2. Results

In this section we have elaborated the different analyses for bibliometric study, well supported by appropriate diagrams and tables. These analyses include trend analysis of publications, types of documents published and the renowned publishers, most contributing journals, most contributing countries, most contributing institutions, most prolific authors and their scientific contribution over time, collaboration network analysis, citations analysis, co-citation and bibliographic coupling of authors, and co-occurrence of keywords.

### 2.1. Trend analysis of publications

**Figure 1:**
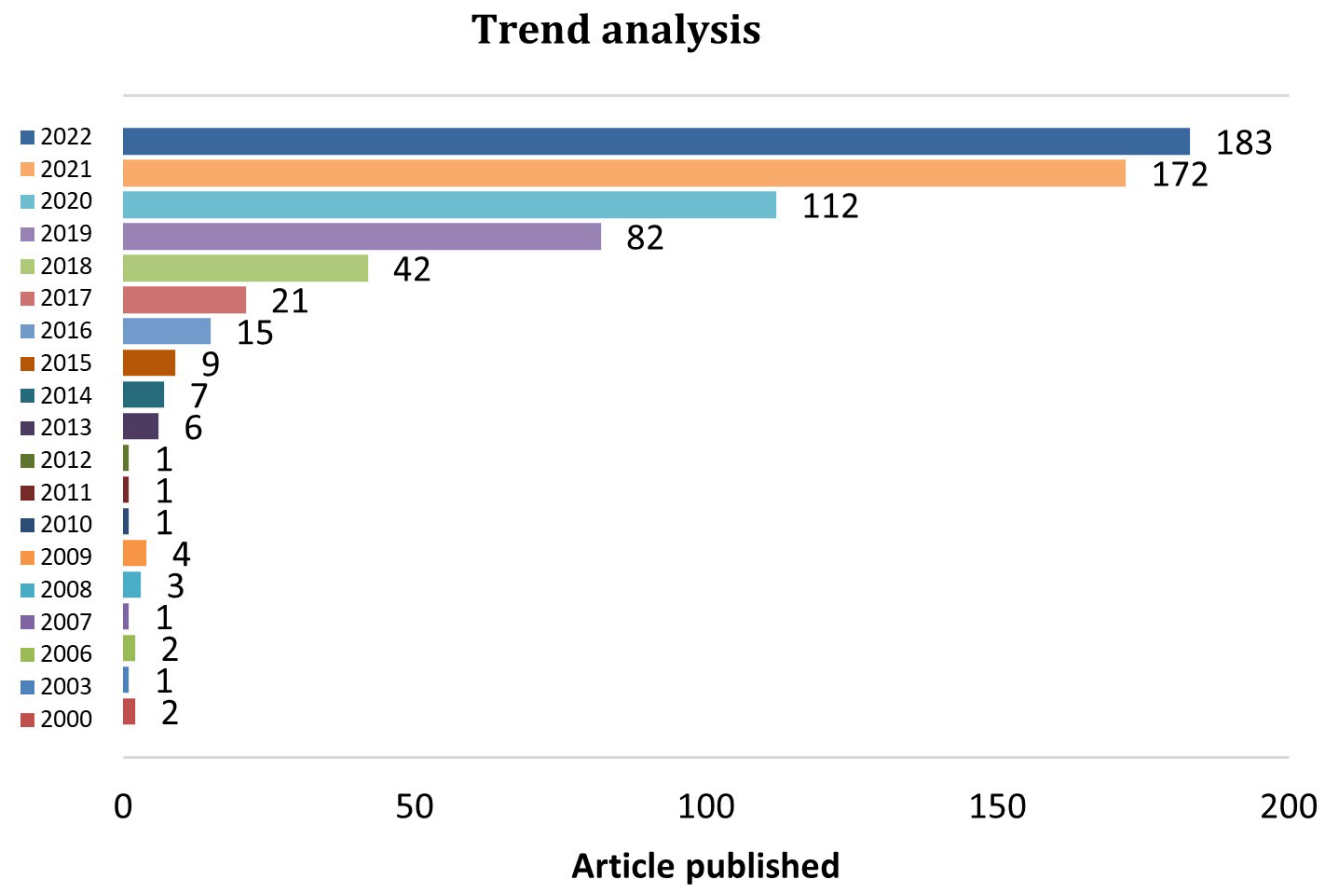
Trend analysis of article published/year.

Figure 1 displays the trends in article production from 2000 to 2022. It was noted that before 2012, there was an average of just 1 study published annually on the topic of machine learning to combat AMR. This is despite the fact that the field’s publication began in 2000. With an average of up to 7 papers published per year between 2013 and 2015, the annual publications showed a relatively slow progression. An inflection point can be seen in 2017, when there were more publications than 20. After that, there were consistently more publications, particularly from 2019 onward, with more than 80% of the papers published in the previous 4 years. Such a growth profile demonstrates how this area is gaining ground and impact in the scientific academia. After 2019, the number of articles published annually quadrupled to 122 on average, reaching a peak in 2022 (183 articles/year). We can deduce that machine learning approaches are becoming increasingly promising in the identification or prediction of AMR and would continue to flourish in the near future based on the trend of growth that has been observed.

### 2.2. Types of documents published and the renowned publishers

**Figure 2:**
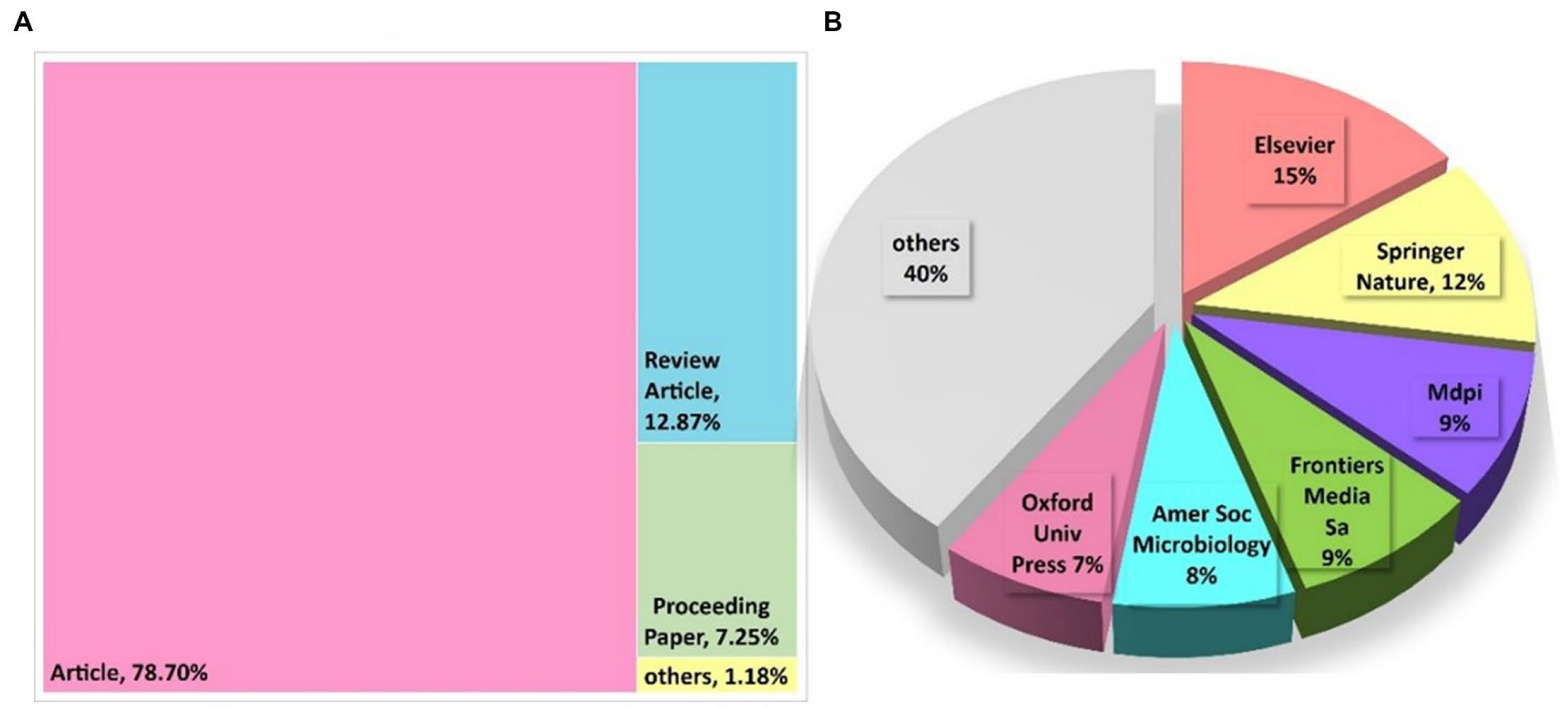
**(A)** Types of articles published. **(B)** Top 6 contributing publishers.

Among the 676 publications that were obtained, the majority of them were empirical papers, constituting 78.7% (532 articles) of the corpus. Review articles made up 12.87% (87 articles), while proceeding papers accounted for 7.25% (49 publications), totaling 98.82% of the publications (as depicted in Figure 2A). Despite a considerable number of review articles published, no bibliometric analysis has been released so far. Presently, out of the 58 publishers, only six have contributed to 60% of the corpus. Among them, Elsevier leads with 15% (104 articles), followed by Springer Nature with 12% (81 articles), MDPI (61 articles), and Frontiers media SA (57 articles), each contributing 9% of all published articles (as illustrated in Figure 2B).

### 2.3. Most contributing journals

The presence of 676 publications in 310 different journals highlights the extensive diversity of literature on machine learning and its application in AMR. As demonstrated in Table 1, the top 10 journals in terms of article count make up 25.14% of the corpus and 21.43% of the total citations. Among these, in terms of publishing output, Frontiers in Microbiology has emerged as the most productive journal with 33 published articles, followed by Scientific Reports (29 articles), PLoS One (17 articles), and Antibiotics (16 articles) respectively. However, in terms of citation impact, Scientific Reports is leading the pack with 591 citations, while Frontiers in Microbiology has received 393 citations, positioning it as the second-most cited journal. If we rank the journals based on their H-index (using https://www.scimagojr.com/journalrank.php), Bioinformatics ranks first with an H-index of 415, PLoS One secures the second position with an H-index of 367, and Antimicrobial Agents and Chemotherapy comes in third with an H-index of 269.

**Table 1:** Top 10 leading journals on the basis of article published.

Journal	Article count	Citation count	Average citation per article (ACPA)	H-index	Publisher
Frontiers in Microbiology	33	393	11.9	166	Frontiers Media SA
Scientific Reports	29	591	20.37	242	Springer Nature
PLoS one	17	281	16.5	367	Public Library of science
Antibiotics	16	52	3.25	47	MDPI
BMC Bioinformatics	15	168	11.2	218	BMC
Briefings in Bioinformatics	15	121	8.06	121	Oxford University Press
mSystems	15	121	8.06	54	American society for Microbiology
Antimicrobial Agents and Chemotherapy	11	239	21.72	269	American society for Microbiology
PeerJ	10	41	4.1	83	PeerJ Inc.
Bioinformatics	9	290	32.22	415	Oxford University Press

### 2.4. Most contributing countries

The total number of articles contributed by 74 different countries is 676, with Table 2 displaying the top 10 countries based on article count. The United States has the highest contribution, with 254 articles, accounting for over 37% of the entire corpus. The United Kingdom (78 articles, 11.53%) and China (103 articles, 15.23%) rank second and third, respectively, in terms of contribution. In addition, the United States has a more considerable global academic impact than any other country, as demonstrated by the highest citation count (5466). Canada ranks second in citations with 1879. Moreover, technologically advanced countries such as the United Kingdom, China, and Germany have made significant contributions, with corresponding citation counts of 1250, 1166, and 1000, respectively.

**Table 2:** Top 10 countries on the basis of article published.

Country	Article count	Total citations	Average citation per article (ACPA)
USA	254	5,466	21.51
China	103	1,166	11.32
UK	78	1,250	16.02
Canada	40	1,879	46.97
Australia	38	543	14.28
Germany	38	1,000	26.31
India	34	340	10
Spain	33	553	16.75
Switzerland	28	469	16.75
Italy	25	383	15.32

### 2.5. Most contributing institutions

A total of 1288 institutions have contributed to the 676 publications, with the University of California participating in the most papers (35). Harvard University (except school of medicine) (29), Harvard Medical School (21), the US Department of Energy (21), and Imperial College London (20) make up the top 5 universities based on article count (Table 3). Harvard Medical School has received the most citations, cited 2148 times, followed by Harvard University (1359) (All schools except Medicine) and the University of California (1310). In terms of average citations per article, Harvard Medical School takes the top position with 102.21, followed by the Massachusetts Institute of Technology with 74.4.

**Table 3:** Top 10 institutions on the basis of article published.

Organization	Article count	Total citations	Average citation per article
University of California	35	1,359	38.82
Harvard University (All schools except Medicine)	29	1,310	45.17
Harvard Medical School	21	2,148	102.21
United States Department of Energy	21	750	35.71
Imperial College London	20	436	21.8
Chinese Academy of Sciences	18	210	11.66
Massachusetts Institute of Technology	15	1,116	74.4
Cornell University	14	312	22.28
University of London	13	278	21.38
University of Oxford	13	243	18.69

### 2.6. Most prolific authors and their scientific contribution over time

**Figure 3:**
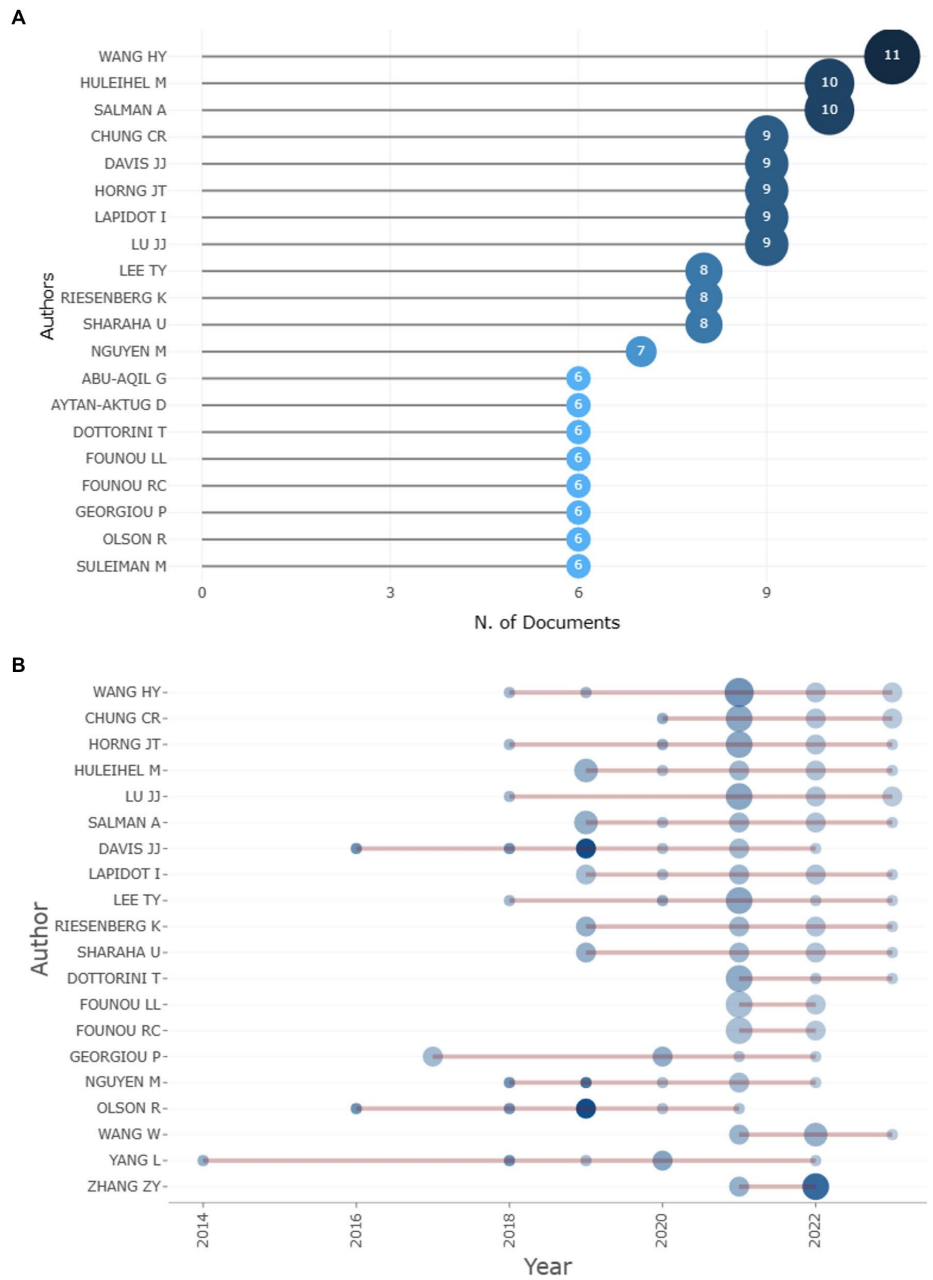
Top 10 authors on the basis of article count and author’s scientific production over time.

A total of 3913 authors have contributed to the 676 articles about the subject of the study. Based on the number of articles published on the topic, the top 10 authors are depicted in Figure 3. Wang, Hsin-Yao is ranked first with 11 articles, while Huleihel, Mahmoud, and Salman, Ahmad follow closely with 10 articles each. Additionally, Lapidot, Itshak; Chung, Chia-Ru; Davis, James J.; Horng, Jorng-Tzong; and Lu, Jang-Jih have contributed 9 publications on machine learning applications in AMR. Looking at the authors’ overall scientific output, Wang, Hsin-Yao, Chung, Chia-Ru, Horng, Jorng-Tzong, Lu, Jang-Jih, and Lee, Tzong-Yi have been the most active authors in the past 2 years, while Huleihel, Mahmoud, and Salman, Ahmad have contributed the majority of articles from 2018 to 20, as shown in Figure 3.

### 2.7. Collaboration network analysis

Extensive collaboration between academics and researchers at all levels is a prominent feature of academic research and is often used as an indicator for evaluating research collaboration (Trotta et al., 2022). The current study aimed to evaluate the degree of intellectual collaboration between authors, institutions, and countries.

#### 2.7.1. Author collaboration

**Figure 4:**
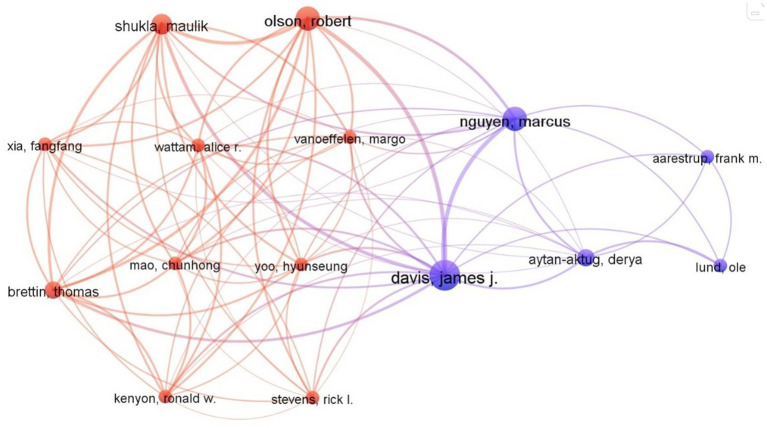
Collaboration network of authors.

The author collaboration network analysis revealed a significant collaboration network consisting of 158 productive authors, with at least three articles each, connected by a total of 83 collaborations and a link strength of 203 (Figure 4). The overall link strength indicates the strength of all collaborations between a particular researcher and other researchers. Davis James J has the highest link strength of 6 and is the closest collaborator to Nguyen Marcus and Olson Robert. The second-most collaborative partners, with a link strength of 5 each, are Shukla Maulik and Davis James J, as well as Shukla Maulik and Olson Robert. Davis James J and Nguyen Marcus have worked with the most authors, a total of 14. Additionally, eight authors, including Shukla Maulik; Olson Robert; Mao Chunhong; Wattam Alice R; Vanoeffelen Margo; Yoo Hyunseung; Aytan-Aktug Derya; and Brettin Thomas, have exhibited strong collaboration networks by collaborating with 12 distinct authors. Furthermore, Xia FangFang and Stevens Rick have each collaborated with 11 authors.

#### 2.7.2. Institutions

**Figure 5:**
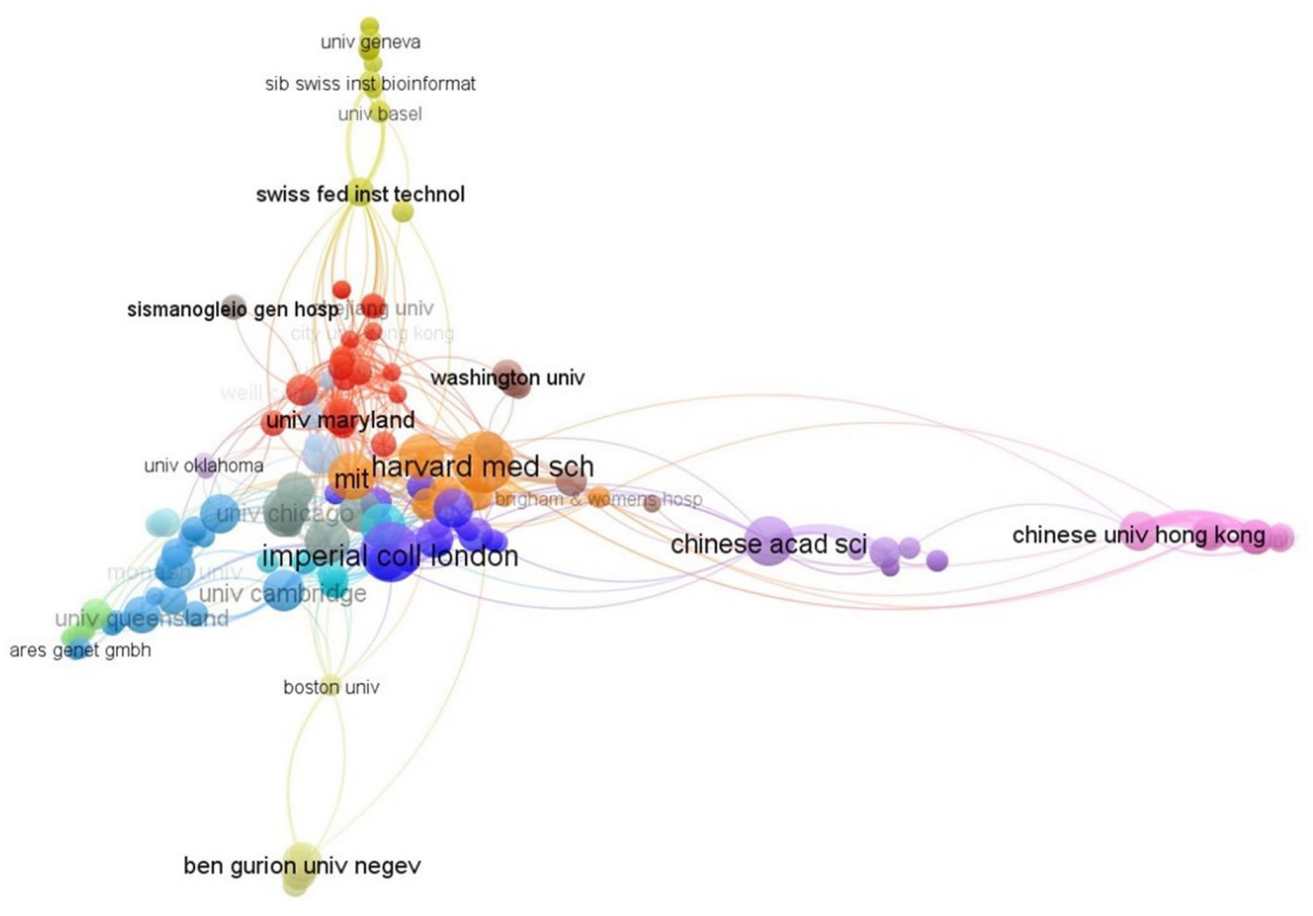
Collaboration network of institutions.

In VOSviewer, the collaboration analysis of research institutions revealed that the largest collaborating network was formed by 158 prominent institutions that had published at least three articles, with a total of 606 linkages and 926 link strength (Figure 5). The Massachusetts Institute of Technology has the highest number of collaborators (37), followed by Weill Cornell Medical College (25), Oxford University (24), Wellcome Sanger Institute, and University of Maryland (22), Harvard University, Pasteur Institute of Korea, University of Minnesota, and Astar (21). The Chinese University of Hong Kong, Asia University, and the Chang Gung Memorial Hospital are the closest partners, each with 7 link strengths. Harvard Medical School has 6 link strengths with the Massachusetts General Hospital and Harvard University.

#### 2.7.3. Countries

**Figure 6:**
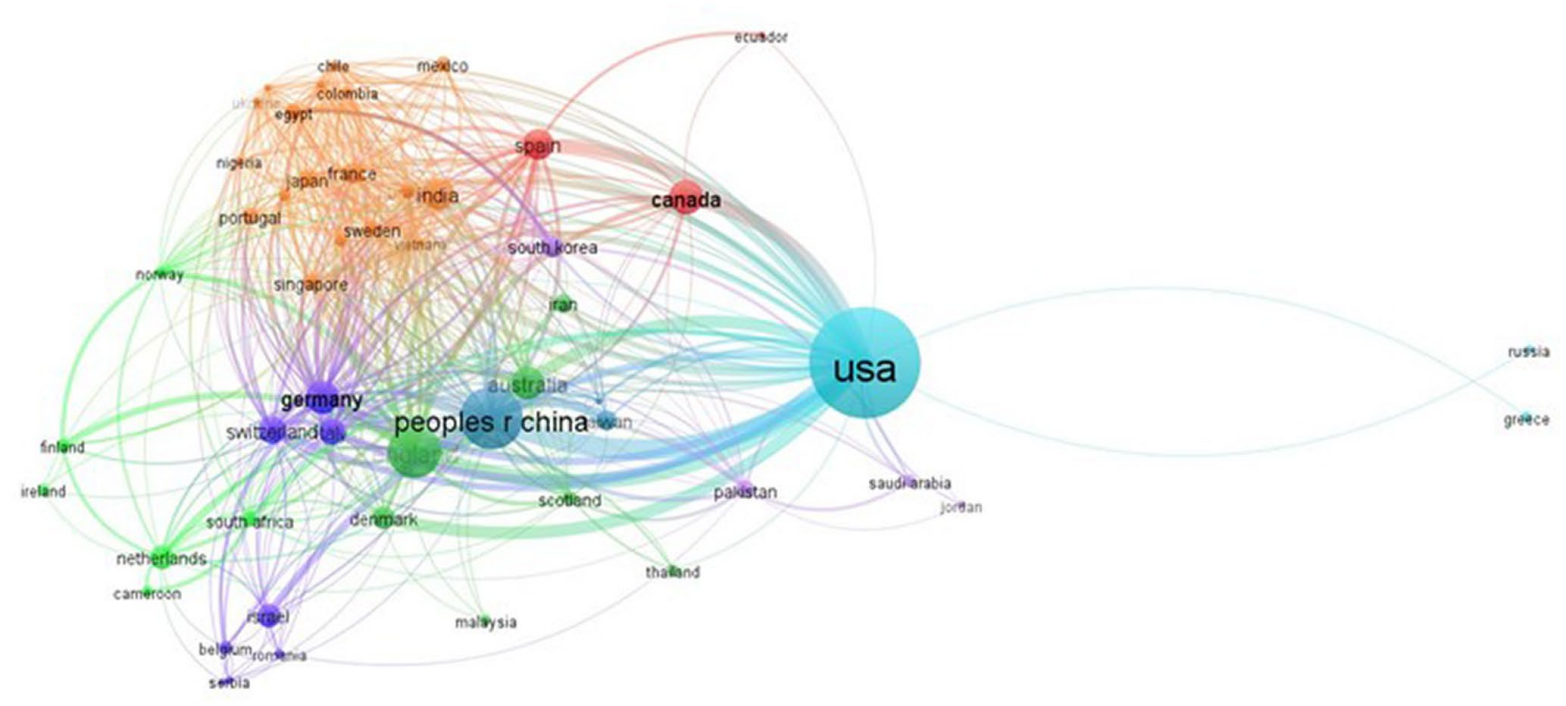
Collaboration network of countries.

In the analysis of collaboration among contributing countries, 52 productive countries with at least three articles each were identified (Figure 6). The United States was found to lead the world in collaborative networks, with the highest number of collaborations with 45 countries. The United Kingdom came in second with 43 collaborators, followed by Germany (42), Italy (40), and China (39). Among the collaborating partners of the United States, China had the highest number of publications with 18, followed by Canada and the United Kingdom with 13 each. The second-most frequent collaborators, with 11 link strengths each, were the United States, Denmark, and Australia. The United States and Spain were third with a total link strength of 10.

### 2.8. Citations analysis

**Figure 7:**
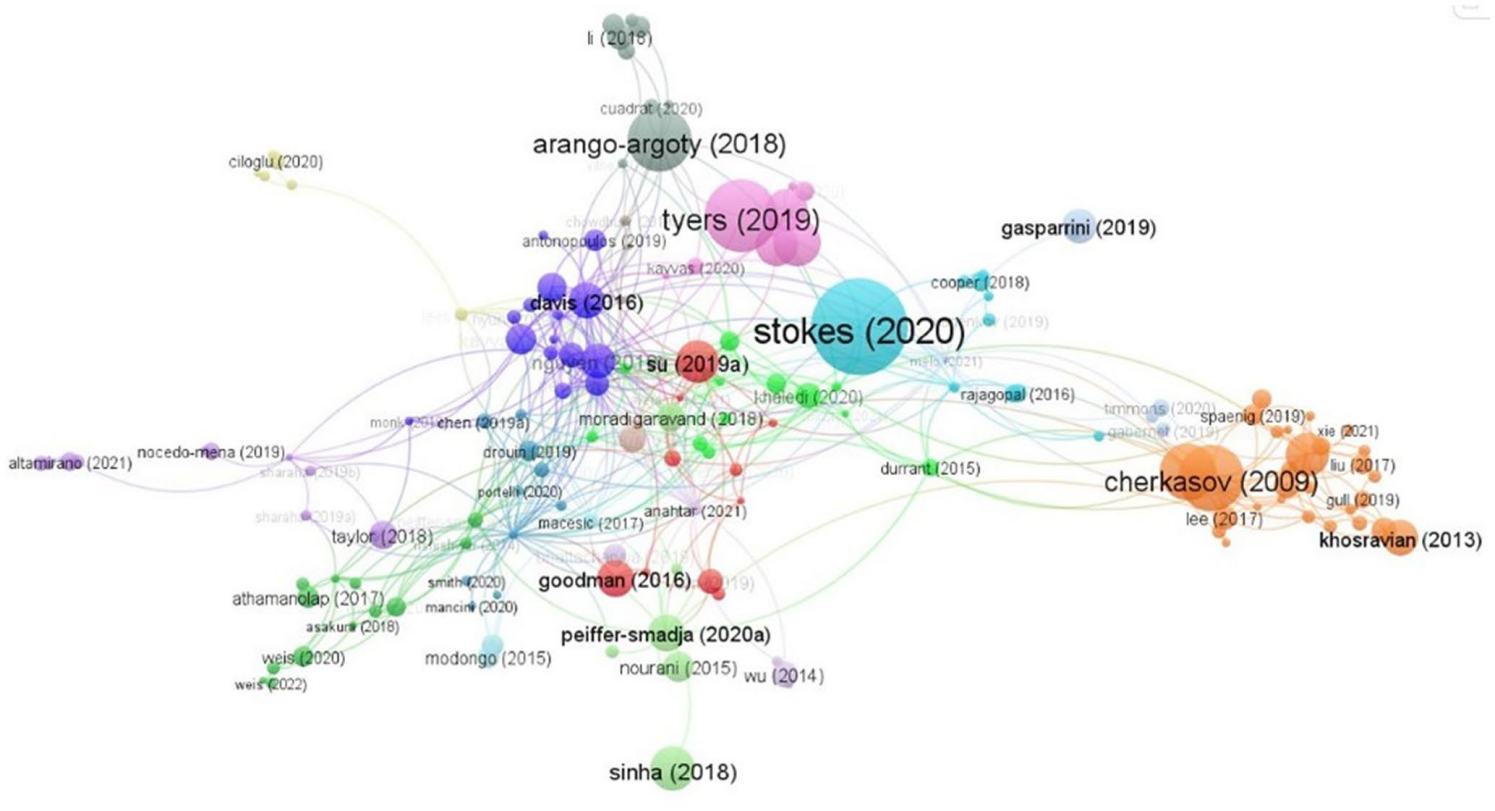
Citation analysis of articles published.

Citation analysis is a technique used to assess the impact and importance of a research work by counting the number of times it has been cited by other authors (Patience et al., 2017). This method is commonly used in bibliometric research to measure the interconnectedness of publications based on their citation relationships. Using VOSviewer for citation analysis, it was found that 246 articles received at least 10 citations, while 26 papers received more than 50 citations, and 21 articles received over 100 citations. The most frequently cited work, with a total of 500 citations, is the empirical study by Stokes et al. (2020). Tyers and Wright (2019), Cherkasov et al. (2009), Arango-Argoty et al. (2018), and Fjell et al. (2009) are the next most cited authors, with 317, 274, 257, and 212 citations, respectively (Figure 7). The top 10 most cited articles are listed in Table 4.

**Table 4:** Top 10 cited articles with their citation count, document type, their source, and country of origin.

No.	Title	Total citation	Article type	journal	Country of first author
1	A deep learning approach to antibiotic discovery	500	Empirical	Cell	USA
2	Drug combinations: a strategy to extend the life of antibiotics in the 21st century	317	Review	Nature	Canada
3	Use of artificial intelligence in the design of small peptide antibiotics effective against a broad spectrum of highly antibiotic-resistant superbugs	274	Empirical	ACS chemical Biology	Canada
4	DeepARG: a deep learning approach for predicting antibiotic resistance genes from metagenomic data	257	Empirical	Microbiome	USA
5	Identification of novel antibacterial peptides by chemoinformatic and machine learning	212	Empirical	Journal of Medicinal Chemistry	Canada
6	Antibiotic-induced alterations of the murine gut microbiota and subsequent effects on colonization resistance against *Clostridium difficile*	167	Empirical	mBio	USA
7	Bacterial metabolism and antibiotic efficacy	160	Review	Cell Metabolism	USA
8	Emerging technologies for molecular diagnosis of sepsis	147	Review	Clinical Biology Reviews	USA
9	Deep learning improves antimicrobial peptide recognition	141	Empirical	Bioinformatics	USA
10	A white-box machine learning approach for revealing antibiotic mechanisms of action	136	Empirical	Cell	USA

### 2.9. Co-citation and bibliographic coupling of authors

**Figure 8:**
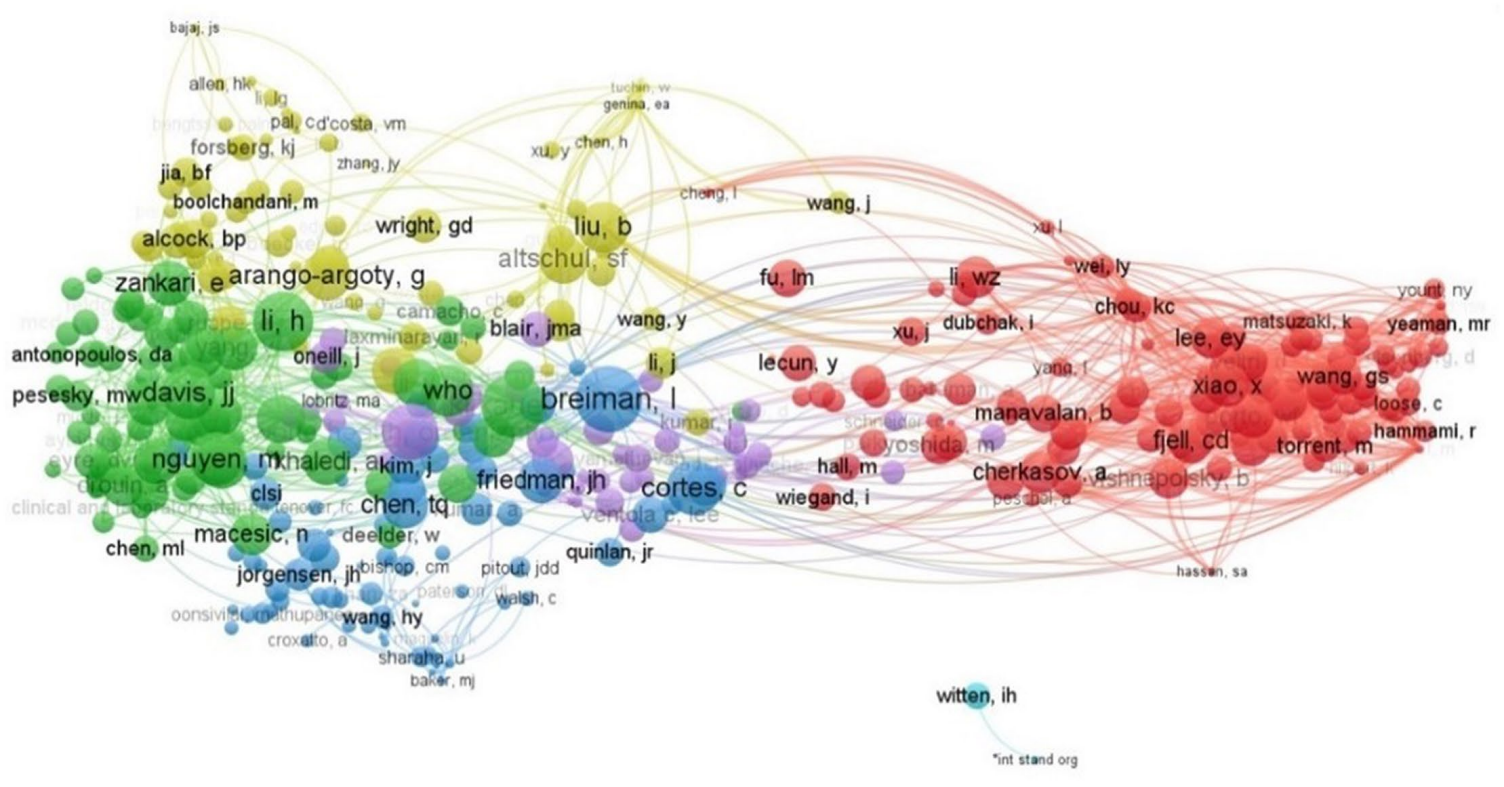
Co-citation analysis of authors.

**Figure 9:**
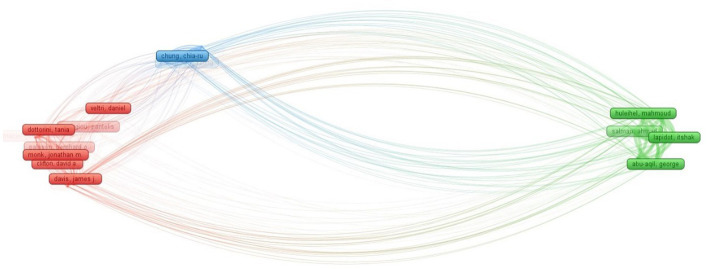
Bibliographic coupling analysis of authors.

Co-citation analysis is a bibliometric method that measures the frequency with which two documents are cited together in other documents. When two documents are cited in the reference list of a third document, they are considered to be co-cited, and the strength of their co-citation relationship indicates their semantic relatedness. Bibliographic coupling, on the other hand, is an alternative method that determines the degree to which two works address the same topic by identifying whether they cite one or more of the same references. The strength of the bibliographic coupling relationship is determined by the number of shared citations between the two works, and it can help researchers identify earlier, relevant, and similar studies (Figures 8–9).

In a co-citation analysis conducted using VOSviewer, it was found that 382 authors were co-cited at least 10 times. Tuchin, W. and Genina, E.A. had the highest number of co-citations, with 216 link strengths, followed by Yount, NY; Lee, E.Y.; and Yeaman, MR; with 85 and 78 link strengths, respectively. Lee EY and Yeaman MR came in fifth place, with a link strength of 72. In the co-citation network analysis, Breiman, I was found to have the most co-cited authors, with 324, followed by Kilkauer T and Li H with 312 and 294 distinct co-cited authors, respectively. As for bibliographic coupling, Huleihel, Mahmoud, and Salman Ahmad had the strongest link strength of 1571, indicating a potential similarity between the two works. Lapidot, Itshak, followed by Huleihel, Mahmoud, and Salman Ahmad, had 1409 link strength each, while Salman Ahmad; Riesenberg, Klaris; and Sharaha, Uraib had a link strength of 1406 each.

### 2.10. Co-occurrence of keywords:

**Figure 10:**
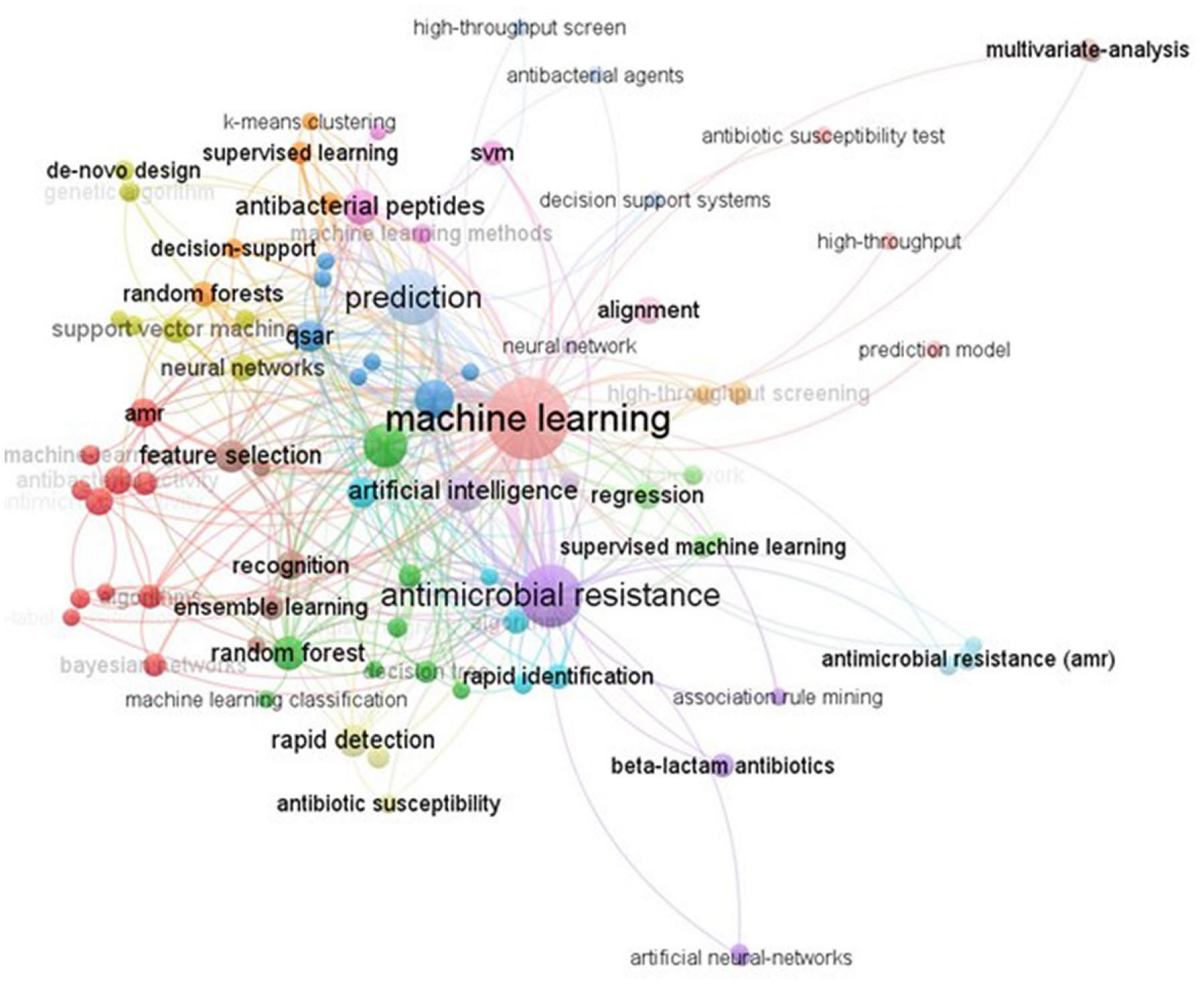
Co-occurrence of total keywords.

The analysis of keyword co-occurrence in a corpus provides insight into the most commonly used keywords and research trends in a particular field, such as the study of AMR prediction through machine learning (Yu et al., 2020; Farhat et al., 2023). Using the VOSviewer program, 3061 keywords were initially identified, but only 83 relevant keywords related to machine learning for AMR prediction were retained for analysis. These keywords were categorized into 18 clusters, with the largest cluster centered around the term “machine learning,” along with other relevant algorithmic terms and specific AMR techniques (see Figure 10). The second-largest cluster focused on machine learning applications for AMR prediction, while the third-largest cluster emphasized various prediction models and techniques.

**Figure 11:**
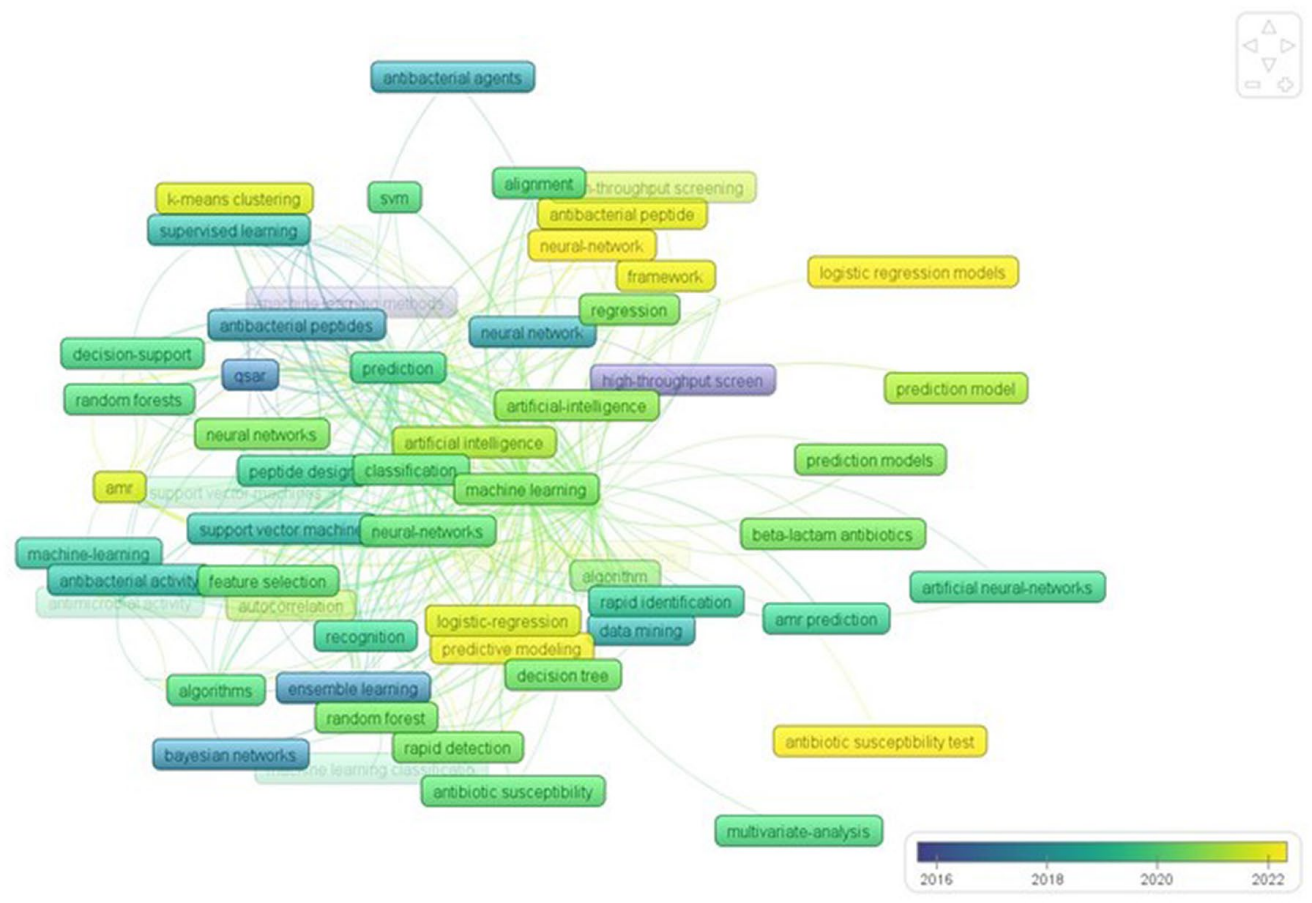
Overlay visualization of keywords.

The overlay visualization of the keyword evolution over time (Figure 11) revealed that older machine learning techniques, such as “supervised learning,” “Bayesian network,” and “support network machine,” were commonly used at the start of the study. However, after 2018, newer techniques like “deep learning,” “artificial neural network,” and “computer-aided drug design” gained popularity, indicating a shift towards improving AMR gene prediction accuracy. These trends suggest that researchers are exploring various methods to combat the growing problem of AMR and develop novel strategies.

## 3. Discussion

In the last 2 years, machine learning has been applied in various healthcare branches (Mufti et al., 2021; Fatima et al., 2022; Naaz et al., 2022; Rashid Irshad et al., 2023) among which the growing problem of AMR is one of the trending research areas (Cockerill, 1999). Traditional antimicrobial susceptibility testing procedures call for isolating bacteria from human specimens using culture techniques, after which the recovered bacteria are subjected to various antibiotic concentrations in numerous assays to determine which concentration limits growth (Wiegand et al., 2008; Nguyen et al., 2018). These methods are time-consuming and expensive (Cockerill, 1999; Wiegand et al., 2008). The fact that the machine learning models gain the AMR information from the data makes them stand out among others that do not require a prior knowledge. Given that the model can be interpreted, scientists can use these models to not only anticipate AMR but also to identify previously unidentified AMR pathways. Machine learning algorithms have just started to be used in the field of AMR from 2000 and until 2006, very discrete publications emerged. It is only after 2016 when the studies are more focused in this particular area.

Bibliometric assessments can assist to forecast future trends in a certain academic topic by providing a high-level overview of its current state (Trotta et al., 2022). Therefore, in this study, we investigated publications on machine learning applications in AMR with a focus on the countries, organizations, journals, authors, and trending keywords related to this subject. A total of 676 journal articles, available in the Web of Science database, were published from 2000 to 2022. The bibliometric data revealed that as of January 2023, 3913 active authors from 74 different countries had contributed to this area of research. There were 1288 organizations involved in publishing these articles in 310 different journals. A tremendous rise in this area of research represents the increasing interest in machine learning application in AMR. Here, we discovered that over 74 countries have made contributions to the field and the number of papers devoted to this topic is increasing every year. In light of these findings, we predict that numerous in-depth research examining various machine learning algorithms to predict AMR in different species using the publicly available databases would be published in the upcoming years.

The topic of applying machine learning in AMR is highly researched, with 676 publications spread out over 310 journals, suggesting a merging of the disciplines of life science and computer science. This is evident from the fact that the journals publishing on this topic belong to both fields. The 310 journals are published by 58 different publishers, but only six of them, namely Elsevier, Springer Nature, MDPI, Frontiers Media SA, American Society for Microbiology, and Oxford University Press, account for 60% of the publications. Notably, the top 10 journals with the most publications are mostly owned by these publishers, except for Elsevier, which is the top publisher but does not feature in the top 10 journals. Among the top 10 journals, the American Society for Microbiology and Oxford University Press each contribute two, while Springer Nature, MDPI, and Frontiers Media SA each contribute one.

Measuring a country’s academic influence can be done by examining the total number of papers produced and the total number of citations it receives. The United States leads the world in academic influence in the field of machine learning and AMR, with 254 publications and 5466 citations, accounting for over 37% of the total corpus. Canada, despite having only the fourth-highest number of articles, received the second-highest number of citations (1879), trailing only the United States. The United Kingdom, China, and Germany have also made notable contributions, with corresponding citation counts of 1250, 1166, and 1000. Collaboration networks are essential in advancing the field of study, and the United States has collaborated with 45 countries, including China, Canada, and the United Kingdom, among others. The United Kingdom, Germany, Italy, and China also have extensive collaborative networks, with 43, 42, 40, and 39 collaborators, respectively, which aids in global information flow.

According to the number of articles, University of California, Harvard University, Harvard Medical School and the US Department of Energy and Imperial College London are the top five organizations engaged in research on the topic. When institutions are examined for collaboration, it is found that the closest partners are either those in the same country or those in nearby countries. Initiatives to forge closer ties could aid this field’s future advancement due to the comparatively low level of collaboration across the institutes. The exchange of information would be improved and advancement in this field of study would be encouraged with more international collaboration.

When the author’s overall scientific output is carried out it is observed that Huleihel, Mahmoud, and Salman, Ahmad have contributed the majority of articles between 2018 to 20 while Wang, Hsin-Yao, Chung (11 articles), Chia-Ru, Horng, Jorng-Tzong, Lu, Jang-Jih, and Lee, Tzong-Yi are the five most active authors from the previous 2 years with 9 articles each. However, Davis James J, Nguyen Marcus, Shukla Maulik, Olson Robert, and others have the strongest network of collaboration.

Keyword co-occurrence analysis paves a way for researchers as they navigate across literature and highlights significant research interests and topics in particular field. An illustration of co-occurrence network of keywords was generated with the relevant topics to machine learning in AMR. The most important and heavily weighted keywords in this network are probably research hotspots of the concerned subject, where there is still a demand for research on these topics and the related research directions. The evolution of keywords through time may be seen by overlay visualization analysis, and it also displays keywords that have received more attention from researchers, indicating they are significant active research areas that need further investigation. The co-occurrence study shows that different machine learning algorithms are being used to either detect AMR or find new alternatives. Many methods, including supervised learning, ensemble learning, Bayesian hyperparameter optimization, quantitative structure–activity relationships (QSAR), and support vector machines (SVM), have been around for a while Stokes et al. (2020).

The top five cited articles highlight different approaches to addressing the urgent need for new antibiotics in the face of increasing antibiotic resistance. For example Stokes et al. (2020), Tyers and Wright (2019), and Cherkasov et al. (2009) are primarily focused at using machine learning techniques to identify novel antibacterial molecules or peptides. Halicin a structurally distinct molecule with broad-spectrum antibacterial activity against resistant pathogens is reported in Stokes et al. (2020). Cherkasov et al. (2009) describes the development of quantitative *in silico* models of antibiotic activity which proved highly effective in predicting the activity of virtual peptides against multidrug-resistant “Superbugs.” Fjell et al. (2009) reports the successful *in silico* screening for potent antibiotic peptides which were found to have activities comparable or superior to those of conventional antibiotics Tyers and Wright (2019) and Arango-Argoty et al. (2018) propose strategies for addressing antibiotic resistance through combinations of antibiotics and non-antibiotic activity-enhancing compounds and through the use of deep learning approaches for comprehensive global monitoring of antibiotic resistance genes in environmental media. Overall, these studies demonstrate the potential of machine learning and other innovative approaches for discovering novel antibiotics and combating antibiotic resistance. Additionally, efforts to curb the overuse and misuse of antibiotics and promote responsible antibiotic stewardship are also crucial in addressing the global challenge of antibiotic resistance

Several machine learning algorithms, including decision support systems, random forest, rapid detection, decision trees, high-throughput screening, and multivariate analysis, have been applied in AMR detection (Schubert et al., 2015; Macesic et al., 2020; Melo et al., 2021; Wang et al., 2022; Yasir et al., 2022). The growth of the machine learning mechanism, through the use of deep learning models such as artificial neural networks, has advanced machine learning-driven AMR research (Cherkasov et al., 2009; Fjell et al., 2009; Arango-Argoty et al., 2018; Popa et al., 2022). Current methods include automated antibiotic discovery, logistic regression models, and k-means clustering (Veltri et al., 2018; Barlandas-Quintana and Martinez-Ledesma, 2020; Valizadehaslani et al., 2020; Marini et al., 2022). These algorithms train the machine learning system to identify unique features and resolve the complicated network, enabling computer-aided drug design in the field of AMR (Sinha et al., 2018; Tyers and Wright, 2019). These findings provide a foundation for identifying current scientific hotspots and guiding future research endeavors.

## 4. Methodology

### 4.1. Data extraction

We used the Web of Science database in January 2023 to find articles with the search queries “antimicrobial resis*” or “antibacterial resis*” AND “machine learning.” The identified articles that contain the search query in the title, abstract, or keywords are chosen for the bibliometric review study. Only English-language journal articles that were published up through January 2023 were included in the search results. Thereafter, 681 articles were retrieved. Then, manual screening was performed to improve the quality of the data by reading the complete texts and looking at the articles’ content to weed out the superfluous ones. Consequently, 676 articles were chosen for the present analysis. The retrieved articles are evaluated using the following criteria: Organization, Countries/Regions, Journals, Total Citations, Number of Publications Per Year, Keywords, and Number of Publications Per Year. The complete records are downloaded for bibliometric analyses and imported into the Biblioshiny (Bibiliometrix) and VOSviewer software (Eck and Waltman, 2010; Aria and Cuccurullo, 2017; Moral-Muñoz et al., 2019).

Various indicators have been utilized in the literature during bibliometric analysis, including total article count, total citation count, total link strength, average citations per article (ACPA), and Hirsch index (H-index). These metrics are commonly used in bibliometric studies, with the H-index being a widely recognized measure of research quality and quantity for authors and research avenues. ACPA is also widely accepted as a measure of research impact for individual works, authors, and publication avenues. Citation and co-citation are useful for exploring the scientific impact and themes of the study under consideration, and co-authorship and co-occurrence have also been investigated to analyze scientific collaboration. All of these indicators have been taken into account in this bibliometric study.

### 4.2. Data analysis

A total of 676 publications from 74 different countries and 1288 different institutions on the topic of AMR and machine learning have been published in 310 different journals. These articles were contributed by 3913 authors, with a total of 10714 citations and 3061 keywords. The pattern of articles published from 2000 to 2022 is shown in a trend analysis of publications for each year. By employing the full counting approach, which only displays the elements related with one another, co-authorship analysis, citation analysis, bibliographic coupling is all carried out. Additionally, co-occurrence of total keywords, author’s keywords, and indexed keywords are also performed to analyze the research trend and trending topics in machine learning approaches used in AMR. Larger circles denote more partnerships and stronger ties between people who collaborate more regularly in various illustration maps of the collaboration network of authors, institutions, and countries while larger circle denotes more citations and links with other co-cited partners in the citation and bibliographic coupling maps. The keyword map using the complete counting method groups related keywords together and gives equal weight to each co-occurrence link, making the circles around the terms with higher frequency larger.

## 5. Conclusion

The present study utilized literature obtained from the Web of Science database to showcase the advancements made in scientific knowledge of machine learning and AMR from 2000 to 2022. The study offers a comprehensive analysis of the existing literature, highlighting significant authors and scientific collaborations, as well as identifying the most common topics and keywords using keyword co-occurrence analysis and overlay visualization. The results provide valuable information on current journals, authors, and extensively explored topics in this field, making it a useful guide for individuals interested in contributing to this discipline. The study also shows a significant increase in the amount of literature on the subject since 2018, primarily due to the introduction of new machine learning algorithms for AMR detection. These advancements have improved the accuracy of methods by utilizing various machine learning techniques and deep learning algorithms. This bibliometric study not only emphasizes the current research directions but also suggests that diverse methods may be feasible in the future, enhancing the predictive efficiency and accuracy of AMR prediction through machine learning.

The present analysis revealed useful information such as:

- Approximately 60% of the publications in the field of machine learning application in AMR are published by Elsevier, Springer Nature, MDPI, Frontiers Media SA, American Society for Microbiology, and Oxford University Press.
- The leading countries in this field are the United States, United Kingdom, and China. The majority of the leading institutes are located in the US and United Kingdom, with the University of California, Harvard University, and Harvard Medical School being the top three.
- The most prolific journals are Frontiers in Microbiology, Scientific Reports, PLoS One and Antibiotics.
- The most contributing authors in this field are Hsin-Yao Wang, Mahmoud Huleihel, and Ahmad Salman.
- The most commonly used algorithms in AMR prediction are Bayesian hyperparameter optimization, quantitative structure–activity relationships (QSAR), and support vector machines (SVM) and logistic regression models.

## Data availability statement

The original contributions presented in the study are included in the article/supplementary material, further inquiries can be directed to the corresponding authors.

## Author contributions

FF: conceptualization, methodology, data curation, and writing—original draft preparation. FF and SS: formal analysis. DM: resources and funding acquisition. MA, SA, DM, and SS: writing—review and editing. All authors contributed to the article and approved the submitted version.

## Conflict of interest

The authors declare that the research was conducted in the absence of any commercial or financial relationships that could be construed as a potential conflict of interest.

## Publisher’s note

All claims expressed in this article are solely those of the authors and do not necessarily represent those of their affiliated organizations, or those of the publisher, the editors and the reviewers. Any product that may be evaluated in this article, or claim that may be made by its manufacturer, is not guaranteed or endorsed by the publisher.
